# Genetics of Ankylosing Spondylitis—Focusing on the Ethnic Difference Between East Asia and Europe

**DOI:** 10.3389/fgene.2021.671682

**Published:** 2021-06-14

**Authors:** Xin Wu, Geng Wang, Luding Zhang, Huji Xu

**Affiliations:** ^1^Department of Rheumatology and Immunology, Shanghai Changzheng Hospital, Second Military Medical University, Shanghai, China; ^2^The University of Queensland Diamantina Institute, The University of Queensland, Brisbane, QLD, Australia; ^3^Department of Health Management, Shanghai Changzheng Hospital, Second Military Medical University, Shanghai, China; ^4^Peking-Tsinghua Center for Life Sciences, Tsinghua University, Beijing, China; ^5^School of Clinical Medicine, Tsinghua University, Beijing, China

**Keywords:** ankylosing spondylitis, genetics, polygenetic risk score, East Asia, Europe

## Abstract

Ankylosing spondylitis (AS) is a common, highly heritable inflammatory arthritis affecting the mainly axial joints in both East Asia and Europe. To date, the pathogenesis of AS is still unknown, although we know that genetics play a vital role in it. The HLA-B27 allele is found in over 85% of AS patients. However, strong evidence suggests that other major histocompatibility complex (MHC) and non-MHC genes are also involved in the pathogenesis. In addition, current data showed that there were significant differences in both genomics and metagenomics among the different ethnic populations. The investigation of the key role of the microbiome in AS pathogenesis also highlighted the host–microbiome genetic interactions. Here, we systematically review current AS genetic research data and further compare genetic differences, especially between East Asian and European groups, which may highlight the challenge in future genetic studies.

## Introduction

Ankylosing spondylitis (AS) is one of the commonest rheumatic diseases in both Asia and Europe. It is a highly heritable chronic inflammatory disease that mainly affects the axial joints, but also has peripheral joints and various organs involvement (Brown et al., 1997, 2000). Pathogenesis of AS is still unknown. The worldwide distribution of AS is closely related to the carrier rate of human leukocyte antigen (HLA)-B27 in population. The prevalence of the disease is about 0.55% of Caucasian population (Braun et al., 1998) and 0.26% in Chinese (Wigley et al., 1994) but less common in Japanese and Africans, mostly attributing to the parallel carrier status of HLA-B27 alleles in these ancestry groups. While the HLA-B27 allele is found in over 85% of patients (Caffrey and James, 1973), there is strong evidence indicating that other major histocompatibility complex (MHC) and non-MHC genes also jointly play roles in the pathogenesis of the disease. Interestingly, current data showed an obvious disease-associated genetic discrepancy across different populations. The observed genetic heterogeneity across divergent populations at several risk loci is by differences in allele frequencies, linkage disequilibrium patterns, effect sizes of associated polymorphisms, or a combination of these factors (International Genetics of Ankylosing Spondylitis Consortium et al., 2013). The objective of this review is mainly to summarize the currently available genetic data and to further make comparisons of this disease genetically between East Asian and European populations.

## The Difference in HLA Alleles

Associations between *HLA-B27* and AS were first reported in 1972 (which were some of the earliest described genetic associations), and it remains the most substantial risk factor for AS (Stokes et al., 1972). There are significant differences in the worldwide distribution of *HLA-B27* and its subtypes (Khan et al., 2007; Gragert et al., 2013). The prevalence of *HLA-B27* positivity in the Chinese and Korean populations has been reported to be from 4 to 8% and 2.3 to 7%, respectively (Kim and Kim, 2010; Yang et al., 2013), which is lower than that in Caucasians but much higher than that in the Japanese population (1%) (Feltkamp et al., 2001).

**FIGURE 1:**
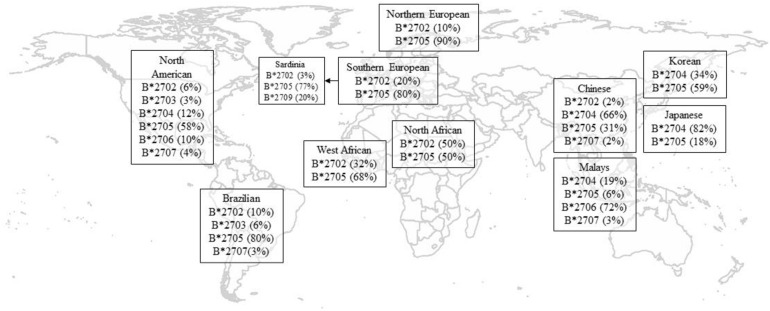
Geographical distribution of HLA-B27 subtypes. Only the most frequent alleles were shown, and statistics are rounded off for simplicity and indicate percentage in healthy controls. Data of North American were adapted from Gragert et al. (2013; Supplementary Table 1). Data for Korean were adapted from Park et al. (2009). Data of other populations were adapted from Khan et al. (2007).

HLA-B27 plays a pivotal role in the pathogenesis of AS. To date, in both Europeans and Asians, the most accurate tag SNP of *HLA-B27* is rs116488202 (International Genetics of Ankylosing Spondylitis Consortium et al., 2013), which is superior to previously reported tag SNP rs4349859 and rs13202464 in Asian populations (Lin et al., 2011). To date, there are 213 known alleles of *HLA-B27* at the nucleotide sequence level, while at the translated protein level, there are 160 known subtypes based on one or more amino acid sequence differences (Khan, 2017). The frequencies of *HLA-B27* alleles vary in different race groups (Supplementary Table 1 and Figure 1; Khan et al., 2007; Gragert et al., 2013). Like other ethnic groups, more than 80% of Chinese AS patients are *HLA-B27* positive, but the primary subtype is *HLA-B^∗^27:04*, followed by *HLA-B^∗^27:05*, which is a predominate subtype for Caucasian cases (Liu et al., 2010). The distribution of *HLA-B27* subtypes also reveals substantial demographic and geographic diversity in China. Although *HLA-B^∗^27:04* is a major subtype in Chinese individuals, it has been reported that the proportion of *HLA-B^∗^27:04* carriers in AS patients were higher in southern China than in northern China, whereas *HLA-B^∗^27:05* positivity was the reverse (Rong and Jieruo, 2013). Being consistent with Mainland Chinese Han Cases, Taiwan Han population is also dominated by *HLA-B^∗^27:04* (Yang et al., 2004; Liu et al., 2010). As for Korea being up north of China geographically, it makes sense that *B^∗^27:05* is the predominant subtype in Koreans, which is similar to Caucasians but different from other Asians down south (Park et al., 2009).

*HLA-B^∗^27:06* has a relatively weak and negative association with AS compared to the *HLA-B^∗^27:04* subtype in Southeast Asia (Nasution et al., 1997; Garcia-Fernandez et al., 2001; Chen et al., 2002), which is more prevalent in Malay descendants (Lopez-Larrea et al., 1995; Gonzalez-Roces et al., 1997; Ren et al., 1997; Hou et al., 2007). In addition, *B^∗^27:09* was found not associated with AS in Sardinia (D’Amato et al., 1995). Mathieu et al. (2008a, b) have proposed a possible evolutionary effect of genetic selection by malaria infection, which could explain the absence of risk haplotypes for AS where malaria was endemic.

Recently, a large-scale study in European case–control cohorts has been initially genotyped by Illumina Immunochip (International Genetics of Ankylosing Spondylitis Consortium et al., 2013), taking the lead in fine-mapping MHC region of HLA classic alleles, SNP, and amino-acid residues in European AS cases and controls. This study suggested the associations with *HLA-B40* and multiple other class I and II alleles (Cortes et al., 2015). It also demonstrated that in that Caucasian the amino-acid sequence of *HLA-B* at position 97 in the B-peptide-binding pocket is the crucial determinant of HLA associations with AS. After controlling for the associated haplotypes in HLA-B, independent associations with variants in the *HLA-A*, *HLA-DPB1*, and *HLA-DRB1* loci were observed.

As for East Asian populations, several loci associated with AS in the Chinese population have been identified, including *HLA-B6*0 and MHC I chain-related gene A (*MICA*) (Ho and Chen, 2013; Zhou et al., 2014). A recent case–control study in Korean additionally identified the association with AS at HLA-C^∗^15:02 (Kim et al., 2015). In our recent study, we analyzed the associations of AS across the MHC aiming to identify potentially causal SNPs, amino acids, or haplotypes using an extended cohort of East Asian ancestry (1,637 Chinese, Taiwanese, and Korean AS cases and 1,589 ethnically matched controls). We have assessed the MHC association of AS in an expanded East Asian cohort. Imputation of the MHC region was conducted in order to assess the variants, HLA classical alleles, and amino-acid residue HLA proteins. This study suggests that the HLA-B associations (B27 and B40) are mainly driven by the amino acids at positions 70 and 97, which locate in the B-pocket of HLA-B peptide-binding groove. Except for HLA-B associations, previously reported East Asian-specific association at *HLA-C^∗^15* had been validated. In addition, a novel association at *HLA-DQB1^∗^04* has been identified (Wang et al., 2020). However, our study needs further validation in larger cohort and conduct with better MHC imputation reference panel of the Pan-Asian population.

## The Difference in non-MHC Genetics

As discussed above, AS is strongly associated with variants in the MHC region and HLA alleles. However, *HLA-B27* and other MHC genes contribute no more than one-third of the genetic risk. It has been intensely investigated that genetic factor non-MHC variants contribute to disease susceptibility. So far, at least 36 genetic variants in non-MHC regions have been identified as associated with AS in genome-wide association study (GWAS) (Brown et al., 2016).

A large-scale multi-ethnic case–control association study performed with Illumina Immunochip microarray provided a new perspective on the similarities and differences in AS susceptibility between East Asian and Caucasians. A total of 13 loci had at least nominal levels of association in East Asians (Chinese, Koreans, Taiwanese), whereas 23 achieved genome-wide significance in white Europeans (International Genetics of Ankylosing Spondylitis Consortium et al., 2013; Ellinghaus et al., 2016; Robinson et al., 2016). Additional studies have been performed in European cohorts. An exome-wide study further identified a novel genome-wide significant association at *CDKAL1*, and several suggestive and secondary loci (Robinson et al., 2016). Ellinghaus et al. (2016) conducted a case–control Immunochip study of five closely associated conditions, including AS, primary sclerosing cholangitis, psoriasis, Crohn’s disease, and ulcerative colitis, in a cohort of European ancestry, delineated the genetic overlap between the conditions, and identified 17 new genome-wide significant susceptibility loci of AS. It also showed that comorbidities between AS and the other chronic inflammatory diseases were mostly attributed to genetic pleiotropy. However, the promising findings of the susceptibility and pleiotropy in Caucasians need to be validated in Asian cohorts.

**FIGURE 2:**
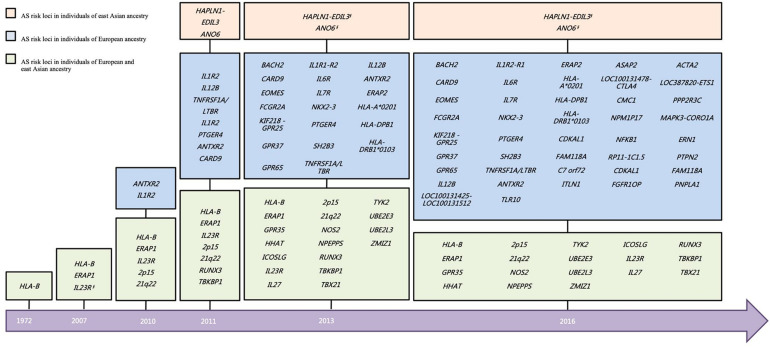
Historical overview of disease susceptibility polymorphisms identified in AS. Risk loci selected with a genome-wide significance threshold of *P* < 5 × 10^–7^ as well as secondary associations (*P* < 5 × 10^–4^ after conditioned for the primary associated SNP), in the previous studies are included. *But different SNP. ^§^No independent support for the two loci in other East Asian cohort. AS, ankylosing spondylitis.

On account of the limited sample size of the East Asian cohort in the Immunochip study, the power of the East Asian cohort was much inferior to that of the European cohort. However, there is hitherto more than 40% overall of the associated loci that have been validated in East Asian (Figure 2), including *ERAP1*, *GPR35*, *HHAT*, *HLA-B*, *ICOSLG*, *IL23R*, *IL27*, *NOS2*, *NPEPPS*, *RUNX3*, *TBX21*, *TYK2*, *UBE2E3*, *UBE2L3*, and *ZMIZ*1, and two intergenic regions (2p15 and 21q22) (Brown et al., 2016). Besides, a GWAS study in Han Chinese identified two AS associated loci (*HAPLN1-EDIL3* and *ANO6*) (Lin et al., 2011). However, few signals of the association have been observed on these two loci in an independent Immunochip study of both East Asians and Caucasians.

Even though most large-scale GWASs have been disproportionately investigated in cohorts of European descent and similar patterns of predisposition were observed between East Asians and Europeans, which is consistent with a shared ancestor origin of the disease-associated SNP, genetic differences exist between ethnic groups pointing to differences in ethnicity-specific etiopathogenesis. Taking the interleukin-23 receptor (IL23R) gene, for example, the primarily associated variants indicated diversity between Europe and Asia. rs11209026, a critical non-synonymous SNP in *IL23R* associated with AS in Caucasians, was not polymorphic in East Asians (Davidson et al., 2009). No other common SNPs on *IL23R* found to be significantly associated with AS in Caucasian European populations. It was suggested that *IL23R* might be a Caucasian-specific associated gene. However, a low-frequency visitant in IL23R, rs76418789, has been reported to potentially attenuate the protective effects of *IL23R* against AS in Han Chinese (Davidson et al., 2013). The same SNP was also nominally associated with AS in Europeans. On the contrary, the minor allele frequency (MAF) of rs76418789 was about 3.7% in East Asians, while only 0.34% in Europeans. In addition to the rare variants on *IL23R*, the SNPs on *STAT3* were also found associated with AS in the Chinese population, which is a downstream molecule of IL-23R in the IL-23 signaling pathway involving the differentiation of Th17 cell populations (Davidson et al., 2011). It may indicate that the sharing effect on Th17 cells is attributed to different mechanisms of disease pathogenesis. Among the other AS-associated loci defined in Europeans but not in East Asians, only the primary associated variant rs17765610 on *BACH2* presents a different frequency of over six times greater in Europeans than in East Asians (East Asians, 1.8%; Europeans, 11.8%) (International Genetics of Ankylosing Spondylitis Consortium et al., 2013). The findings might indicate that the different associations between East Asia and Europe were not attributed to the various frequencies of associated variants at most loci.

## Metagenomics

It has been drawing increasing attention that gut inflammation plays a pivotal role in the pathogenesis of AS. IBD, as a paradigm for microbiota effects on the pathogenesis of the immune-mediated disease, is strongly related and significantly overlaps with AS in genetic predisposition. HLA-B27 transgenic rats did not develop gut and joint inflammation when bred in a germ-free environment, while inflammations present when they were exposed to healthy gut bacteria (Rath et al., 1996).

Several studies have investigated how the microbiome as a key role in driving the pathogenesis of AS. Intestinal dysbacteriosis may affect the permeability of the intestinal wall, the expression of related inflammatory factors, the intestinal mucosal immune status in AS patients, and molecular mimicry of HLA-B27 (Ebringer and Ghuloom, 1986; Ciccia et al., 2009, 2014, 2015; Wright et al., 2016). Intestinal flora imbalance can also mediate host metabolism and immune function imbalance *via* a series of cytokines, thus participating in the pathogenesis and progress of AS.

Studies have revealed several notable differences in bacterial species and in abundance. Costello et al. (2015) have compared the terminal ileum microbial communities in AS patients with healthy controls in Australia, and Lachnospiraceae, Veillonellaceae, Ruminococcaceae, Rikenellaceae, Bacteroidaceae, Porphyromonadaceae, and Prevotellaceae were significantly enriched or decreased in abundance in AS patients. Wen et al. (2017) have reported a quantitative metagenomics study suggesting that discrete gut microbial signature is associated with the pathogenesis of AS in Chinese, suggesting consistent findings with the report of Costello, such as Prevotellaceae. It also showed some discordance with previous reports in Europeans, like Bacteroidetes, and identified other novel biomarkers that might be involved in the development of AS in the Chinese population (Wen et al., 2017). To further investigate the roles of microbiome in AS pathogenesis, Yin et al. (2019) conducted a case–control metagenomic analysis of 250 Han Chinese. In addition to confirmation of previously reported gut dysbiosis and species differences in AS, the results also indicate that treatment with TNF inhibitor (TNFi) normalizes the gut microbiome. The AS gut microbiome is enriched for bacterial peptides that have previously been shown to be presented by HLA-B27, and that this enrichment is also normalized by TNFi treatment. Bacterial peptides presented by HLA-B27 have been found enriched in gut microbiome in AS patients, which is also normalized by TNFi treatment. Relative to untreated patients, TNFi therapy of AS patients was also associated with a reduction of potentially arthritogenic bacterial peptides, which are enriched bacterial peptides homologous to HLA-B27-presented epitopes. Host-bacteria genetic interactions were also observed between an AS-associated SNP (*RUNX3*) and microbiome, highlighting a non-MHC host genotype influencing AS *via* the microbiome potentially (Yin et al., 2019).

Over the last few decades, Asia, especially China, has experienced rapid urbanization, resulting in massive changes of dietary habits, which directly links to the changes of the microbiome (Yang et al., 2016). Gut microbiota is both an important therapeutic target for AS and an essential biomarker for disease surveillance. The reconstruction of gut microbiota has a potential therapeutic effect on AS patients, and the relationship between intestinal flora and AS deserves further study.

## Clinical Benefits and Prospects

To date, clinical practice data show that the average diagnostic delay of AS is 6–10 years and early treatment, such as anti-TNFα biological agents, have been proved to improve disease outcomes. So, the early prediction of AS is challenging, causing noteworthy. However, the genome-wide significant associated SNPs only represent a trivial fraction of total heritability. A polygenic risk score (PRS) is aiming to use genotype data from thousands of genetic variants to quantify an individual’s genetic risk for specific diseases and has potential as a diagnostic and screening test for common heritable diseases, including AS (Torkamani et al., 2018). In our recent research (Li et al., 2021), we used GWAS data from 15,083 AS cases and 20,902 controls and then developed and validated PRS in European-descent and East Asian ethnicity subjects. PRS showed greater discriminatory capacity and accuracy than HLA-B27 testing, MRI scanning, or CRP testing, and could be used to assist in diagnosing AS among chronic back pain patients, as well as screening populations to identify subjects at increased risk of the disease. When the PRS was derived and tested in individuals of primarily their ancestries, the area under the curves (AUC) of the European and East Asian GRSs were 0.92 and 0.95, respectively. The discriminant capacities were attenuated cross-ethnic validations (AUC = 0.79 for European model in the East Asian cohorts, AUC = 0.88 for East Asian model in the European cohorts). It suggests that the performance of the PRS does vary between ethnic groups. The PRS developed specifically for the East Asian population performed considerably better in that population than did the European PRS, elucidating the different genetic landscapes between East Asian and European.

The similarities and differences in the genetic features of AS between East Asia and other ethnic populations have demonstrated the utility of gene mapping in probing the genetic diversity among different ancestry groups. It is of great importance to confirm the associated loci in populations of different ancestries, which is a crucial indicator of the overall significance of defining the true disease-causing variant. The identification of genetic signatures in both the East Asian and European populations will provide additional details for unraveling the genetic basis of AS and other autoimmune diseases.

PRS will be of clinical use, particularly for a disease of low prevalence and high heritability, like AS. Given the low cost of microarray, even next-generation sequencing, PRS makes it possible for population screening. Modified PRS models of ethnic specificity and multi-omics, including epigenomics and metagenomics, need more comprehensive training cohorts and more accurate evidence for better disease prediction of AS. To date, most genetics studies have been undertaken in European ancestry. Therefore, further studies of multi-omics analyses, microbiota, and environmental factors will require undertaking or expanding trans-ethnic cohorts.

## Author Contributions

XW, GW, LZ, and HX made substantial contributions to draft and revise the manuscript.

## Conflict of Interest

The authors declare that the research was conducted in the absence of any commercial or financial relationships that could be construed as a potential conflict of interest.
