# Supplementary material for: Genetics of Ankylosing Spondylitis—Focusing on the Ethnic Difference Between East Asia and Europe
Source: Front Genet. 2021 Jun 14;12:671682. doi: 10.3389/fgene.2021.671682 (PMC8236852; doi:10.3389/fgene.2021.671682)
Supplement: Supplementary file 1 [file Table_1.docx]

**­­­Supplementary Table 1 Allele frequencies of HLA-B27 and subtypes of volunteer donors in different race groups from the National Marrow Donor Program (NMDP) of US (ref.** **Gragert et al., 2013)**

|  | African American | African | South Asian Indian | North American Indian | Caribbean black | Caribbean Indian | European Caucasian | Filipino | Japanese | Korean | Middle Eastern or North Coast of Africa | Mexican or Chicano | Chinese | Hispanic – South or Central American | Southeast Asian | Vietnamese | Total | Percentage of HLA-B27 subltypes |
| --- | --- | --- | --- | --- | --- | --- | --- | --- | --- | --- | --- | --- | --- | --- | --- | --- | --- | --- |
| 2N* | **833162** | **57114** | **370782** | **71582** | **66656** | **230748** | **2485780** | **101228** | **49164** | **155168** | **141780** | **552470** | **199344** | **293428** | **55956** | **87080** | **5751442** |  |
| Allele | **Frequency** | **Frequency** | **Frequency** | **Frequency** | **Frequency** | **Frequency** | **Frequency** | **Frequency** | **Frequency** | **Frequency** | **Frequency** | **Frequency** | **Frequency** | **Frequency** | **Frequency** | **Frequency** | **Frequency** |  |
| B*27:02 | **0.036%** | **0.046%** | **0.077%** | **0.275%** | **0.044%** | **0.352%** | **0.393%** | **0.015%** | **0.018%** | **0.006%** | **0.589%** | **0.195%** | **0.007%** | **0.190%** | **0.093%** | **0.000%** | **2.335%** | **5.81%** |
| B*27:03 | **0.331%** | **0.438%** | **0.002%** | **0.000%** | **0.375%** | **0.026%** | **0.007%** | **0.000%** | **0.000%** | **0.000%** | **0.039%** | **0.025%** | **0.000%** | **0.047%** | **0.002%** | **0.001%** | **1.292%** | **3.21%** |
| B*27:04 | **0.002%** | **0.001%** | **0.198%** | **0.014%** | **0.014%** | **0.003%** | **0.001%** | **0.660%** | **0.203%** | **0.227%** | **0.027%** | **0.001%** | **1.910%** | **0.003%** | **0.554%** | **1.134%** | **4.949%** | **12.31%** |
| B*27:05 | **0.843%** | **0.589%** | **0.828%** | **5.451%** | **0.668%** | **1.722%** | **3.725%** | **0.315%** | **0.226%** | **2.487%** | **1.475%** | **2.216%** | **0.346%** | **1.603%** | **0.711%** | **0.236%** | **23.442%** | **58.30%** |
| B*27:06 | **0.027%** | **0.012%** | **0.009%** | **0.001%** | **0.000%** | **0.001%** | **0.001%** | **2.357%** | **0.006%** | **0.008%** | **0.001%** | **0.012%** | **0.095%** | **0.009%** | **0.406%** | **1.077%** | **4.024%** | **10.01%** |
| B*27:07 | **0.006%** | **0.019%** | **0.506%** | **0.022%** | **0.015%** | **0.052%** | **0.034%** | **0.009%** | **0.004%** | **0.008%** | **0.192%** | **0.024%** | **0.074%** | **0.032%** | **0.399%** | **0.022%** | **1.418%** | **3.53%** |
| B*27:08 | **0.002%** | **0.007%** | **0.000%** | **0.014%** | **0.003%** | **0.010%** | **0.009%** | **0.001%** | **0.000%** | **0.000%** | **0.004%** | **0.009%** | **0.000%** | **0.020%** | **0.000%** | **0.000%** | **0.079%** | **0.20%** |
| B*27:09 | **0.001%** | **0.002%** | **0.001%** | **0.001%** | **0.002%** | **0.000%** | **0.006%** | **0.002%** | **0.000%** | **0.000%** | **0.016%** | **0.002%** | **0.000%** | **0.002%** | **0.000%** | **0.000%** | **0.034%** | **0.08%** |
| B*27:10 | **0.000%** | **0.000%** | **0.000%** | **0.001%** | **0.000%** | **0.000%** | **0.009%** | **0.000%** | **0.000%** | **0.000%** | **0.003%** | **0.004%** | **0.000%** | **0.000%** | **0.000%** | **0.001%** | **0.018%** | **0.04%** |
| B*27:12 | **0.002%** | **0.002%** | **0.000%** | **0.003%** | **0.011%** | **0.010%** | **0.007%** | **0.001%** | **0.000%** | **0.001%** | **0.028%** | **0.008%** | **0.000%** | **0.010%** | **0.000%** | **0.000%** | **0.080%** | **0.20%** |
| B*27:14 | **0.003%** | **0.000%** | **0.000%** | **0.038%** | **0.002%** | **0.000%** | **0.009%** | **0.000%** | **0.000%** | **0.000%** | **0.003%** | **0.001%** | **0.001%** | **0.000%** | **0.000%** | **0.000%** | **0.057%** | **0.14%** |
| B*27:15 | **0.000%** | **0.000%** | **0.000%** | **0.001%** | **0.000%** | **0.000%** | **0.000%** | **0.002%** | **0.000%** | **0.000%** | **0.000%** | **0.000%** | **0.013%** | **0.000%** | **0.000%** | **0.003%** | **0.019%** | **0.05%** |
| B*27:20 | **0.000%** | **0.000%** | **0.000%** | **0.000%** | **0.002%** | **0.000%** | **0.000%** | **0.000%** | **0.000%** | **0.024%** | **0.000%** | **0.000%** | **0.000%** | **0.000%** | **0.000%** | **0.001%** | **0.027%** | **0.07%** |
| B*27 | **2.508%** | **2.238%** | **1.622%** | **5.828%** | **1.134%** | **2.179%** | **4.204%** | **3.364%** | **0.458%** | **2.762%** | **2.382%** | **2.499%** | **2.464%** | **1.916%** | **2.170%** | **2.477%** | **40.21%** | **/** |

Only total alleles with frequency > 0.01% are shown in the table. *N row indicates the number of typed samples; 2N indicates the number of typed alleles.
